# Sinensetin regulates age-related sarcopenia in cultured primary thigh and calf muscle cells

**DOI:** 10.1186/s12906-019-2714-2

**Published:** 2019-10-28

**Authors:** Jin-A Kim, Seong Min Kim, Sang Eun Ha, Preethi Vetrivel, Venu Venkatarame Gowda Saralamma, Eun Hee Kim, Gon Sup Kim

**Affiliations:** 1 Research Institute of Life science and College of Veterinary Medicine, Gyeongsang National University, Jinju, 52828 Republic of Korea; 2 Department of Physical Therapy, International University of Korea, Jinju, 52833 Republic of Korea; 3 Institute for Women’s Health Care, Jinju, 52818 Republic of Korea

**Keywords:** Sarcopenia, Sinensetin, Myogenin, MyoD

## Abstract

**Background:**

Sarcopenia, the decline of skeletal muscle tissue attributed to primary aging is a major concern in older adults. Flavonoids might have potential benefits by modulating the regulation of satellite cells, thus preventing muscle loss. Sinensetin (SIN), a citrus methylated flavone with anti-inflammatory and anti-proliferative activity, can enhance lipolysis. The objective of the present study was to investigate whether SIN might have sarcopenia-suppressing effect on satellite cells from thigh and calf muscle tissues of young and old rats.

**Methods:**

Primary muscle cells were obtained from thigh and calf tissues of young and old group rats by dissection. Obtained satellite cells were incubated with indicated concentrations of SIN (50 and 100 μM) treated and untreated condition in differentiation medium. Morphological changes of cells were examined using a phase-contrast microscope. Protein expression levels of myoD and myogenin were analyzed by Western blot. Cells treated with or without SIN under differentiation condition were also immunocytochemically stained for myogenin and 4′,6-diamidino-2-phenylindole (DAPI).

**Results:**

Morphologically, the differentiation extracted satellite cells was found to be more evident in SIN treated group of aged rat′s cells than that in SIN untreated group. Expression levels of myoD and myogenin proteins involved in myogenesis were increased upon treatment with SIN.

**Conclusions:**

Collectively, our results indicate that SIN can alleviate age-related sarcopenia by increasing differentiation rate and protein levels of myoD and myogenin.

## Background

The process of aging is associated with a continues loss of muscle mass and strength leading to a condition known as sarcopenia in human and animal models [1]. Sarcopenia is one of age-related syndromes encompassing muscle loss related to impaired mobility, chronic disease, and malnutrition. It is a condition caused by devaluation of muscle fiber satellite cells and portrayed by atrophy of type II muscle fibers with aging. The decline in the regenerative capacity by the reduction of satellite cells causes loss of type II fiber skeletal muscle. It affects type I fibers to a lesser extent [2]. The deficit of muscle mass and its impaired function are caused by a sequence of convoluted factors including accumulation of miss-folded, cross-linked, and aggregated molecules and denaturation, causing catastrophic effects on quality and quantity of muscles [3]. Activation of satellite cells is involved in muscle regeneration. This process is coordinated by the expression of several myogenic regulatory factors (MRFs), including myf5, mrf6, myoD, and myogenin. These MRFs are involved in nuclear transcription and they are expressed consecutively during myogenesis [4]. In the mechanism of muscle repair, proteins myoD and myogenin play vital roles in both early and late stages of myogenesis. Protein myoD regulates the activation of satellite cells and proliferation of myoblasts, whereas myogenin engages in the differentiation of these cells [5].

Sarcopenia results from convoluted and interdependent pathophysiological mechanisms that include aging, resistance to postprandial anabolism, neuromuscular compromise, insulin resistance, oxidative stress, mitochondrial dysfunction, and inflammation [6]. Previous reports have suggested that the primary arbitrator of skeletal muscle depleting is systemic inflammation that occurs in accordance with diseases such as chronic obstructive pulmonary disease (COPD), acquired immune deficiency syndrome (AIDS), and cancer [7]. Chronic inflammation results in loss of muscle strength, reduction of muscle mass, and poor functionality. It affects both muscle synthesis and breakdown of proteins through several signaling pathways, leading to sarcopenia. Aging is generally associated with a chronic state of slightly elevated plasma levels of pro-inflammatory mediators, such as nuclear factor kappa B (NF-κB), interleukin 6 (IL-6) and tumor necrosis factor- α (TNF-α) [8].

Herbal products and its components exhibit anti-inflammatory effects by targeting pro-inflammatory mediators involved in multiple cellular signaling pathways [9]. Flavonoids are natural polyphenolic compounds widely distributed in plant parts. Earlier studies have already demonstrated that they possess anti-inflammatory effects by targeting multiple regulatory mechanisms [10–12]. Polymethoxyflavones (PMFs) are ubiquitous in citrus plants. They are of special interest because of their biological effects including anti-inflammatory, anti-cancer, and anti-oxidative activity [13–15]. PMFs have been shown to exhibit anti-inflammatory activity in several inflammation-induced models by suppressing the production of pro-inflammatory cytokine TNF-α, prostaglandin E2 (PGE2), interleukin-1β (IL-1β), and IL-6 by regulating NF-κB pathway [16, 17]. Previous study have suggested that infliximab, a TNF-α inhibitor, can suppress NF-κB activation and reverse the condition of inflammatory mediated sarcopenia in patients with Crohn’s disease [18]. These reports strongly suggest that finding novel therapeutics from natural sources can recover the condition of sarcopenia. Sinensetin (SIN) is one such polymethoxyflavone with anti-oxidant, anti-cancer, and anti-inflammatory effects. It might have a beneficial effect against sarcopenia bececause its anti-oxidant, anti-cancer, and anti-inflammatory effects are intimately correlated with its beneficial actions against various metabolic diseases such as insulin resistance, muscle atrophy damage, and cancer [19–21]. The ability of SIN to alter cellular inflammatory status could be particularly useful for treating sarcopenia. It has been shown that SIN can inhibit LPS-induced inflammation by suppressing the expression of COX-2, iNOS, IL-1β, IL-6, and TNF-α genes associated with inflammation in macrophages [20].

Considering these correlations of SIN with anti-inflammatory effect, we hypothesized that SIN might be able to prevent sarcopenia and improve the function of satellite cells. Thus, the objective of this study was to investigate whether SIN could suppress aging-related sarcopenia using satellite cells isolated from muscle tissues of 6 weeks (Young) and 12 months (Old) Sprague-Dawley rats, an outbred multipurpose breed of albino rat used extensively in medical research especially in sarcopenia [22].

## Methods

### Dissection of skeletal muscle tissues

The life expectancy of SD rats ranges from 30 to 36 months. Six month old rats are equivalent to 18 year old human beings and 24 month old rats are equivalent to 60 year old humans [22]. Male Sprague-Dawley rat were purchased from Samtaco Inc. (Seoul, Republic of Korea). Based on age, they were grouped into 6 weeks (Young) and 12 months (Old). Five rats were chosen for each age group and maintained to obtain stabilization. After 10 days of stabilization, rats were sacrificed to isolate muscle tissues in the laboratory. Satellite cells were isolated from the thigh and calf region. Both muscle tissues were combined and taken for this study. These isolated muscle tissues were initially weighed. Their mass was calculated in comparison with the whole body weight. Rats were anesthetized with diethyl ether via the respiratory route for approximately 2 min in a transparent acrylic jar. All experiments were performed in compliance with relevant laws and guidelines. Animal experiments protocol were reviewed and approved by the Ethical Committee of Gyeongsang National University.

### Isolation of satellite cells and their differentiation

Isolation and differentiation of satellite cells were done as described previously [23]. Briefly, isolated muscle tissues were prepared by washing with 1X phosphate-buffered saline (PBS) followed by washing with Dulbecco′s modified Eagle′s medium (DMEM) from Gibco (BRL Life Technologies, Grand Island, NY, USA). Skeletal tissues were separated from tendons. Adipose tissues remained in isolated whole muscle cells. These satellite cells were treated with 0.1% pronase from Streptomyces griseus (Merck, Darmstadt, Germany) prepared in DMEM medium followed by filteration with a 0.22 μm filter (SPL Life Sciences, Pocheon, Republic of Korea). The solution of pronase was pre-warmed at 37 °C before filtering. Tissue content was centrifuged at 3000 rpm for about 10 min. The supernatant was discarded and the cell pellet was obtained. These cells were cultured in collagen-coated plates (SPL Life Sciences, Pocheon, Republic of Korea) of 1 young group and 3 old groups. These cells were grown up to 80% confluence in proliferation medium containing DMEM supplemented with 20% fetal bovine serum (FBS), 10% horse serum (HS), and 1% penicillin/streptomycin (P/S) purchased from Gibco (BRL Life Technologies, Grand Island, NY, USA). After that, cells were subcultured in differentiation medium containing DMEM, 2% FBS, and 1% P/S. Once the satellite cells were isolated, they were cultured in proliferation medium for 5 days. After reaching 80% confluence, cells were subcultured and maintained for 5 days to stabilize. After stabilization, cells were treated with 10, 25, 50 and 100 μM of sinensetin (SIN) for 5 days in differentiation medium. The diameter and differentiation ability of satellite cells obtained from old group (old rat’s satellite cells) after treatment with 50 and 100 μM SIN were increased in comparison with those of untreated old group of cells. After that, old group cells were grouped into three: 1 group of untreated and 2 groups of treatment with SIN at 50 and 100 μM for 5 days in the differentiation medium.

### Morphological change and measurement of myoblast length

Satellite cells cultured in the differentiation medium were taken for morphological examination under a light microscope (X200) before and after SIN treatment to characterize differentiation based on morphology. The diameter of myoblast was examined and its length upon differentiation was measured using Image J software (U.S. National Institutes of Health, Bethesda, MD, USA).

### Western blot analysis

Western blot analysis was performed to determine levels of myogenic regulatory proteins involved in the activation of satellite cells and regeneration. Briefly, after 5 days of incubation with or without SIN in differentiation medium, cells were lysed with radio immunoprecipitation assay (RIPA) buffer to obtain proteins. Protein concentrations were measured using a Pierce™ BCA protein assay kit (Thermo Scientific™, Waltham, MA, USA), in accordance with the manufacturer’s protocol. Proteins (20 μg) were separated by 8–12% sodium dodecyl sulfate (SDS)-polyacrylamide gels and transferred onto a polyvinylidene difluoride (PVDF) membranes. Blots were then blocked with 5% bovine serum albumin (BSA) solution for 1 h at room temperature and then incubated with primary antibodies [α-tubulin (1:5000), myoD (1:1000), and myogenin (1:1000), Cell Signaling Technology, Danvers, MA, USA] at 4 °C overnight. Membranes were washed within Tris-buffered saline containing 1% Tween 20 (TBS-T, pH 7.4) and then probed with their appropriate horseradish peroxidase-conjugated secondary antibody (1:1000) for 3 h at room temperature. Bands were visualized using Clarity™ ECL substrate reagent (Bio-Rad, Herules, CA, USA) and quantified with ImageJ software program (U.S. National Institutes of Health, Bethesda, MD, USA). Densitometry readings of bands were normalized to the level of α-tubulin as a loading control. Alpha-tubulin is used as a housekeeping protein expressed in skeletal muscle studies. Five samples were measured for each group. Data are presented as representative bands. Statically significance was determined using analysis of variance (ANOVA) followed by a Dunnett’s test (#*p* < 0.05 vs. young, *p < 0.05 vs. old with no SIN).

### Immunofluorescence assay

Differentiated satellite cells were stained immunocytochemically for myogenin. Briefly, cells were cultured on glass coverslips in 12-well plates and incubated with or without SIN in differentiation medium for 5 days. Cells were washed with 1X PBS, suspended in 1X PBS, dried, and fixed with 4% paraformaldehyde in 1X PBS (pH 7.4) for 10 min at room temperature. These cells were then incubated with 1X PBS containing 0.25% Triton X-100 at room temperature for about 10 min and then washed with 1X PBS three times (5 min each time). After that, cells were incubated with 1% BSA at room temperature for 30 min to block unspecific binding. Diluted myogenin antibody was then added to these cells and incubated at 4 °C overnight. After incubation, cells were washed with 1X PBS for 3 times (5 min each time) and treated with secondary antibodies (Alexa Fluor® 488 Conjugate, Thermo Scientific™, Waltham, MA, USA) followed by incubation at room temperature for 1 h in the dark. Counterstaining of cells was performed after decanting antibodies and washing with 1X PBS for 3 times. Cells were stained with a 4′,6-diamidino-2-phenylindole (DAPI, Vectashield H-1500; Vector Laboratories, Inc., Burlingame, CA, USA) for 3 min and washed with 1X PBS. After washing, slides were mounted with coverslips, sealed, and examined under a fluorescence microscope.

### Statistical analysis

All statistical analyses were performed with paired Student’s t-test (two-tailed) and Dunnett’s test using GraphPad Prism 5 program (GraphPad Software Inc., San Diego, CA, USA). Results are expressed as mean ± standard error of the mean (SEM) of at least 3 independent experiments. Statistical significance was set at *p* < 0.05.

## Results

### Young rats have greater muscle weights than old rats

**Fig. 1:**
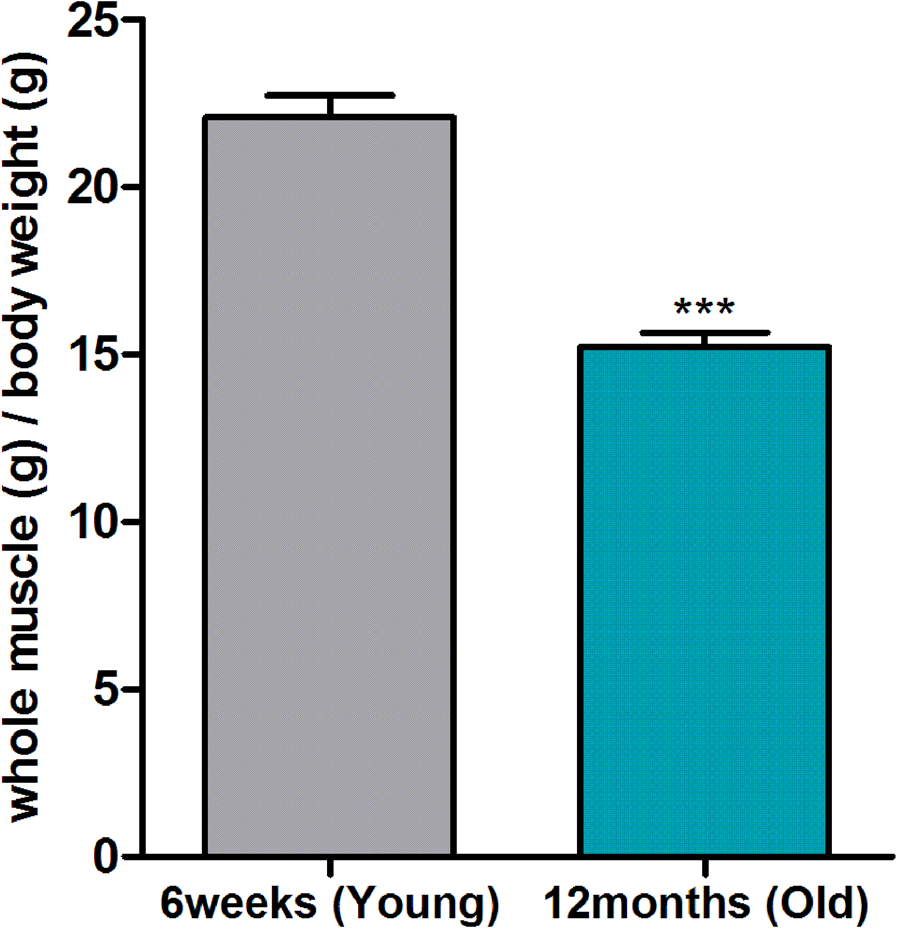
Comparison of thigh and calf skeletal muscle weight between young (6 weeks) and old (12 months) rats. After stabilization for 10 days, young and old groups were sacrificed to obtain thigh and calf skeletal muscles. Muscle weights were divided by body weights. We utilized 5 animals for each group. Data are presented as means ± SEM. Statically significance was determined by Student’s t-test; ***p < 0.001

To compare the muscle weight of thigh and calf region in young and aged rats, 6-week-old young rats and 12-month-old old rats were obtained. After dissecting thigh and calf muscles of young and old rats, whole muscle weight relative to body weight was measured, respectively. Results showed that muscles of young group rat’s weighed more than those of old group rat’s (Fig. 1). Student’s t-test and Dunnett’s test were utilized for statistical comparison between the young and old group rat’s. These muscles were used for the isolation of satellite cells and their differentiation in this study.

### Sinensetin (SIN) treatment enhances myoblast differentiation of satellite cells

**Fig. 2:**
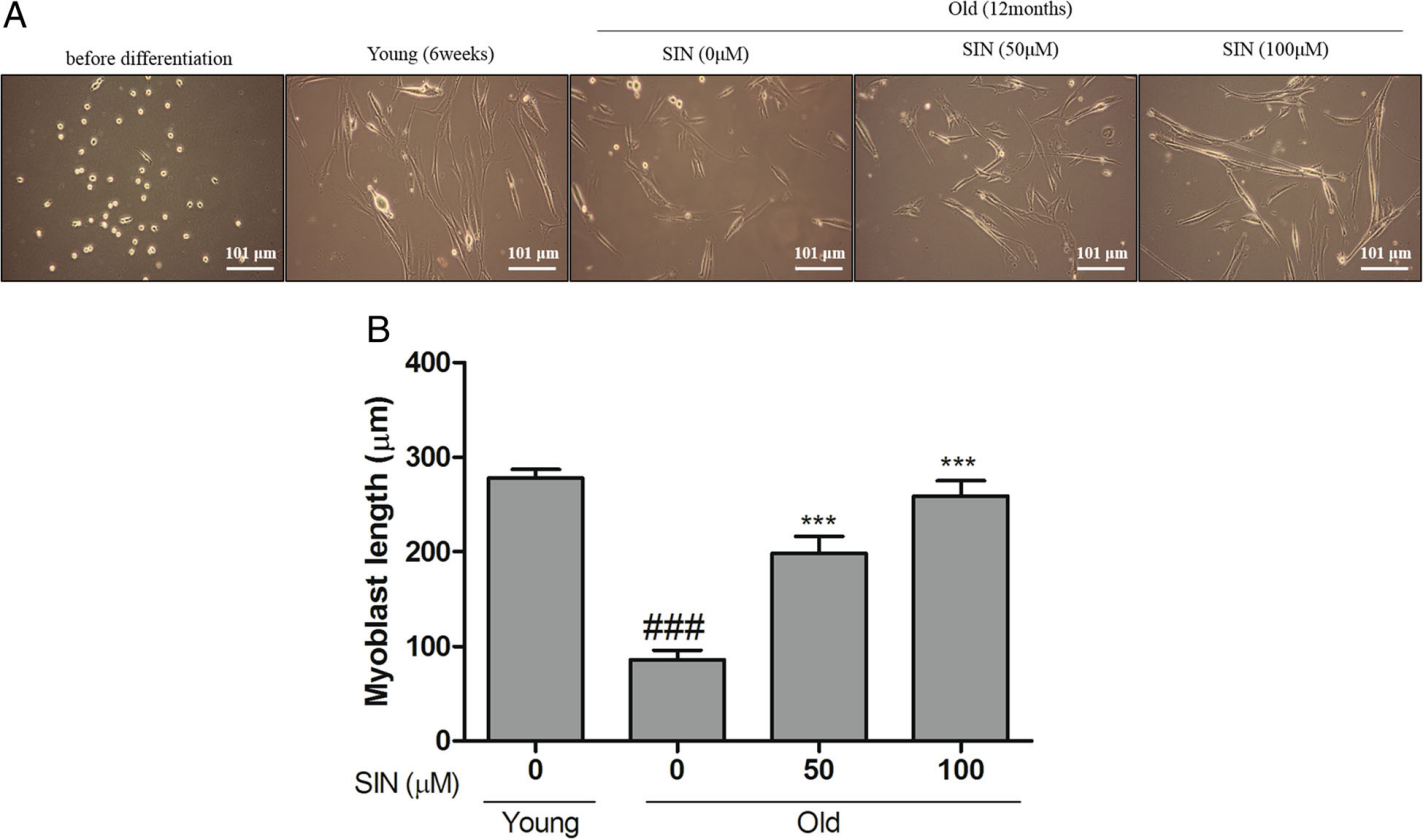
Morphology changes of isolated satellite cells. Isolated satellite cells were cultured in proliferation medium for 5 days. Differentiation medium was then provided to both young and old groups. Old group cells were treated with sinensetin (SIN) at indicated concentrations in differentiation medium. **a** Morphological changes of satellite cells were observed after 5 days of treatment with SIN at indicated concentrations (50 and 100 μM). The observation was done using a light phase-contrast microscope (X200). **b** Myoblast length was measured using Image J software (U.S. National Institutes of Health, Bethesda, MD, USA). Data are expressed in graphical representation with improved length at indicated concentrations of SIN. Data in graphs are presented as mean ± SEM. Statically significance was determined with ANOVA followed by a Dunnett’s test; ###*p* < 0.05 vs. young, ***p < 0.05 vs. old with no SIN

In this study, ex vivo studies was performed using primary satellite cells obtained from thigh and calf muscles of young and old rats. After satellite cells were isolated, they were cultured in proliferation medium for 5 days. After reaching 80% confluency, cells were subcultured and maintained for 5 days to stabilize. Initially, four different concentrations (10, 25, 50 and 100 μM) of SIN were used to treat satellite cells in differentiation medium (data not shown). Morphological observations were used to characterize skeletal muscle cells and the purity of isolated satellite cells. Based on the effect of SIN on satellite cells differentiation by observing morphological changes, 2 concentrations (50 and 100 μM) were adopted for further experiments. To evaluate the effect of SIN on satellite differentiation, satellite cells from old rats were treated with 2 concentrations of SIN (50 and 100 μM) for 5 days consequently in the differentiation medium. Cell differentiation was observed to be more evident in the old group cells upon SIN treatment than that in the untreated control old group cells (Fig. 2a). Ex vivo results suggested that the diameter of myoblast and the differentiation ability of satellite cells in old rats treatment with SIN were significantly increased compared to those in the untreated old group cells (Fig. 2b). It was also observed that the morphology of old group cells was similar to that of young group (young rat’s satellite cells) upon treatment with SIN. Based on this morphological evidence, 50 and 100 μM concentrations of SIN were used for further experiments. There was no significant difference in cell differentiation between young and old group cells treated with SIN. Treatment with 50 and 100 μM SIN showed no significant difference in cell differentiation either statisticaly.

### SIN treatment increases protein levels of myoD and myogenin

**Fig. 3:**
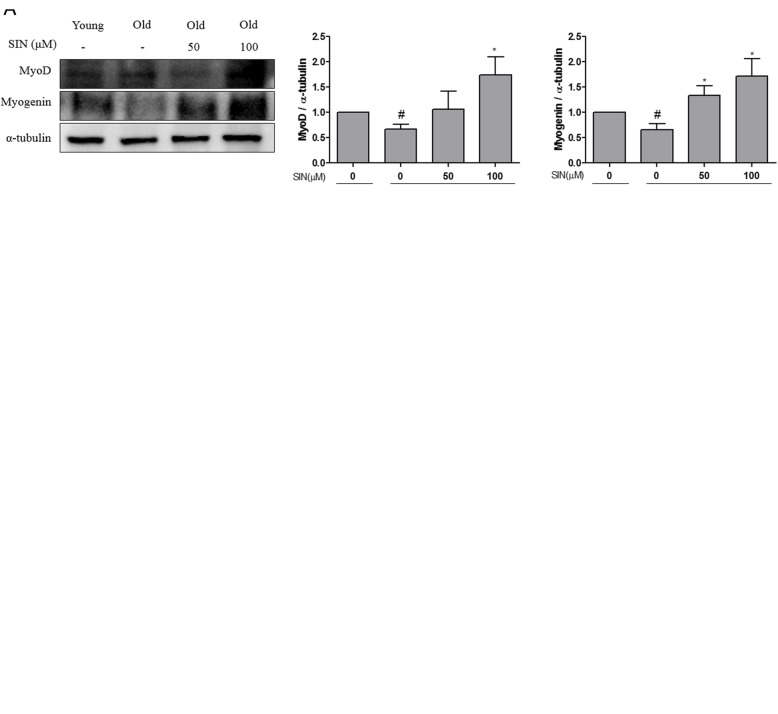
Effect of sinensetin (SIN) on satellite cells of old rats in vitro. After SIN treatment with differentiation medium for 5 days, (**a**) satellite cells were harvested. MyoD and myogenin protein levels were examined by Western blotting. The level of α-tubulin was used for normalization using Image J program. Data in graphs are presented as mean ± SEM. Statically significance was determined with ANOVA followed by a Dunnett’s test; #*p* < 0.05 vs. young, *p < 0.05 vs. old with no SIN. **b** Differentiated satellite cells were immunohistochemically stained for myogenin in green and DAPI in blue. These images were captured with a confocal microscope (X1,000)

Protein levels of myoD and myogenin after differentiation of satellite cells for 5 days with or without SIN treatment were then determined by Western blot analysis. Results showed that protein expression levels of both myoD and myogenin were significantly increased in old group cells after treatment with SIN 50 and 100 μM when compared with untreated old group cells (Fig. 3a). Additionally, myogenin immunofluorescence intensity in differentiated satellite cells was determined. We stained myogenin of satellite cells in the differentiation medium with Alexa Fluor 488 (Green) and nuclei with DAPI (Blue). Results revealed that fluorescence intenstiy of myogenin was increased in SIN treated old group cells compared to that in the untreated old group cells (Fig. 3b). Collectively, these ex vivo analysis results revealed that SIN treatment promoted differentiation and increased expression levels of myoD and myogenin as differentiation marker proteins in SIN treated old group. Similarly, immunofluorescence results showed that myogenin levels were higher in SIN treated old group cells compared to those in the untreated group and that their expression pattern was similar to that in the young group cells.

## Discussion

The primary aim of this study was to determine effects of SIN on satellite cells isolated from skeletal muscle using ex vivo study to reflect the time course of human sarcopenia. Sarcopenia is recognized as a major clinical problem for older people. Research on sarcopenia is expanding tremendously. Age-related deficiency in the number of satellite cells is induced by the loss of skeletal muscle mass and strength [23, 24]. In the current study, separated thigh and calf muscles of young and old rats were weighed and young rats were found to have higher muscular weight than old rats. This indicates that old rates are undergoing loss of muscle mass, a hallmark of sarcopenia.

Acute inflammation models are widely applied to study molecular mechanisms that link inflammation and muscle protein metabolism to sarcopenia [25]. The key role of TNF-α in chronic inflammatory disease is evidenced by potent anti-inflammatory effects of TNF-α antagonist on arthritis and age related sarcopenia. Recent studies have focused on bioactivities of polyphenols and flavonoids against inflammation-related sarcopenia [22, 26]. SIN is one such bioactive flavonoid that possesses anti-inflammatory and chemosensitizing effects equipotent to verapamil and cyclosporin A. Its effect is at least 10 to 100 fold superior to those of other flavonoids such as hesperidin, quercetin, and ferulic acid [19, 20]. Thus, in this study, we focused on the effect of SIN on sarcopenia in rat satellite cells. Results of the current study revealed that cell differentiation was higher in the old group cells upon SIN treatment compared to that in the the untreated old group cells. Such increase in differentiation could be due to increased skeletal muscle mass known to accelerate the regeneration of injured skeletal muscles in old rats [27]. MyoD and myogenin are products of genes belonging to the myoD family expressed by differentiation of myoblasts to root canal cell [28]. MyoD is a myogenic transcription factor that is expressed in proliferating or differentiating stem cells, which are destined towards a myogenic lineage [29]. Myogenin is expressed in satellite cells during differentiation, whereas myoD can be expressed in satellite cells in late proliferation/early differentiation [30]. In the current study, we found that protein levels of myoD and myogenin were increased in SIN treated old rats satellite cells group compared to those in the old rats satellite cells without SIN treatment. Furthermore, myoblast length was also significantly increased with increasing SIN concentration, suggesting the improvement of myoblast differentiation in old group cells. Collectively, these data indicate that the differentiation of cells in SIN treated old group cells is increased compared to that in the untreated old group cells. Previousl studies have also shown that polyphenols can elevate muscle regeneration and myogenic differentiation in mammalian models [31, 32]. These results suggest that SIN may contribute to satellite differentiation in old rat muscles.

**Fig. 4:**
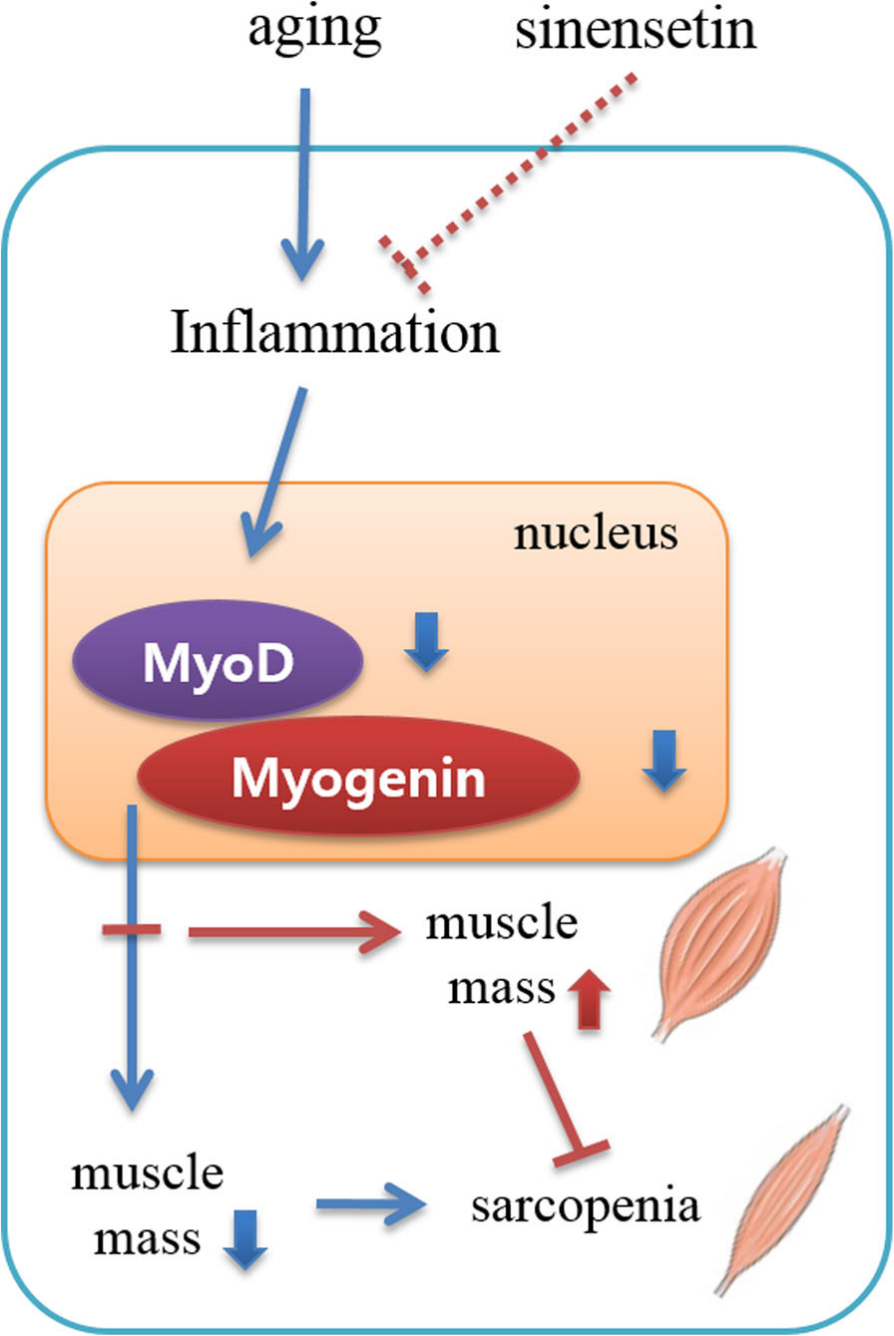
Schematic diagram showing the predicted sinensetin (SIN) pathway for sarcopenia

Our previous study has shown that SIN can suppress lipopolysaccharide (LPS)-induced inflammation through suppression of NF-κB in L6 skeletal muscle [33]. Based on this previous evidence, we anticipate that the increase in protein levels of myoD and myogenin might be through inhibition of inflammation. Based on these significant outcomes of the previous and the current study, the role of SIN in the elevation of myoD and myogenin proteins is briefly summarized in Fig. 4.

## Conclusions

In conclusion, from this study, the differentiation of the satellite cells in old rats group has been recovered similar to that of young rats group upon treatment with SIN. Similarly, the protein levels of myoD and myogenin are increased in satellite cells from SIN treated old rats group compared with the untreated group. These findings suggest that SIN treatment may have a potent effect on the prevention of age-related sarcopenia by increasing the protein levels myoD and myogenin and further detailed study will help to validate the involvement of inflammation. Furthermore, these data provided basic evidence on the effect of SIN on sarcopenia and thus it may be beneficial to produce potent drug against the conditions of muscle loss.

## Data Availability

We have included the necessary data supporting our claims for publication and we are not wishing to disclose the raw data due to confidential issue. (Data will make available on demand).

## Abbreviations

DAPI: 4′,6-diamidino-2-phenylindole
DMEM: Dulbecco′s modified Eagle′s medium
IL-6: Interleukin 6
LPS: lipopolysaccharide
MRFs: Myogenic regulatory factors
NF-κB: Nuclear factor kappa B
PBS: Phosphate buffered saline
SIN: Sinensetin
TNFα: Tumor necrosis factor α
